# Supplementary material for: Cloud BioLinux: pre-configured and on-demand bioinformatics computing for the genomics community
Source: BMC Bioinformatics. 2012 Mar 19;13:42. doi: 10.1186/1471-2105-13-42 (PMC3372431; doi:10.1186/1471-2105-13-42)
Supplement: Additional file 1 — Supplementary 1 Cloud BioLinux software documentation in the form of a mini, self-contained website. Users need to download and uncompress the .zip file, and open through a web browser the "index.html" file available on the main directory. (ZIP 1823 kb). [file 1471-2105-13-42-S1.ZIP › Cloud-BioLinux-Package-Documentation/docs/Rqtl.html]

Bio-Linux Software Documentation Pages

Back to search form

## Rqtl

|  |  |
| --- | --- |
| Name | Rqtl |
| Description | **Rqtl** is part of the QTL Cartographer suite of programs.  **Rqtl** will translate a genetic model or simulate a random model for use by Rcross to simulate a data set. It places a specified number of QTLs (Quantitative Trait Loci) on the molecular map created or translated by Rmap. For simulations, they are placed randomly on the map, and the additive, dominace and epistatic effects are also determined. |
| Homepage | http://statgen.ncsu.edu/qtlcart/Rqtl.php |
| Remote Documentation | http://statgen.ncsu.edu/qtlcart/Rqtl.php |

Will translate a genetic model or simulate a random model to simulate a data set.
